# Impaired Recognition of Static and Dynamic Facial Emotions in Children With Autism Spectrum Disorder Using Stimuli of Varying Intensities, Different Genders, and Age Ranges Faces

**DOI:** 10.3389/fpsyt.2021.693310

**Published:** 2021-08-20

**Authors:** Selima Jelili, Soumeyya Halayem, Amal Taamallah, Selima Ennaifer, Olfa Rajhi, Mohamed Moussa, Melek Ghazzei, Ahmed Nabli, Sami Ouanes, Zeineb Abbes, Malek Hajri, Radhouane Fakhfakh, Asma Bouden

**Affiliations:** ^1^Department of Child and Adolescent Psychiatry, Razi Hospital, Manouba, Tunisia; ^2^Faculty of Medicine, Tunis El Manar University, Tunis, Tunisia; ^3^Department of Psychiatry- Hamad Medical Corporation, Doha, Qatar

**Keywords:** autism, social cognition, facial emotions, emotion recognition, children

## Abstract

A multitude of research on facial emotion recognition (FER) in Autism Spectrum Disorders (ASD) have been published since several years. However, these studies have mainly used static high intensity stimuli, including adult and/or children facial emotions. This current study investigated FER in children with ASD using an innovative task, composed of a combination of static (114 pictures) and dynamic (36 videos) subtests, including children, adolescent, and adult male and female faces, with high, medium, and low intensity of basic facial emotions, and neutral expression. The ASD group consisted of 45 Tunisian verbal children, and the control group consisted of 117 tunisian typically developing children. Both groups were aged 7–12 years. After adjusting for sex, age, mental age, and school grade, the ASD group scored lower than controls on all tests except for the recognition of happiness and fear in the static subtest, and the recognition of happiness, fear, and sadness in the dynamic subtest (*p* ≥ 0.05). In the ASD group, the total score of both the static and the dynamic subtest were positively correlated with the school grade (*p* < 0.001), but not with age, or mental age. Children with ASD performed better in recognizing facial emotions in children than in adults and adolescents on videos and photos (*p* < 0.001). Impairments in FER would have negative impact on the child's social development. Thus, the creation of new intervention instruments aiming to improve emotion recognition strategies at an early stage to individuals with ASD seems fundamental.

## Introduction

Facial expressions of emotion convey non-verbal cues for interferences with others. They allow us to understand motivations and intentions of others. Consequently, they are considered as one of the foundations of social interaction (1).

Over the decades since the 1970s, psychological studies have established that there were six universal basic emotions: sadness, disgust, anger, surprise, happiness, and fear (2–4). Izard (5) suggested that, at the age of six, typically developing individuals are able to recognize several facial emotional expressions, while other studies mentioned that this ability is only totally developed before adolescence (6–8). However, the aptitude to accurately identify emotions is not distributed uniformly across children and may negatively impact social interactions.

Autism spectrum disorders (ASD) are “neurodevelopmental disorders characterized by early-onset qualitative impairments in social interaction, verbal and non-verbal communication, associated with restricted and repetitive interests and behaviors” (9). As social cognition is particularly implicated in ASD, a large number of research have examined facial emotion recognition (FER) in this population over the last decades (10, 11). Recent research in ASD have shown that the ability to recognize facial emotion in children with ASD is not always associated with their intelligence (12, 13), contrarily to NT individuals in whom performance IQ could predict emotion recognition performance in children (14).

Several studies have focused on the assessment of basic emotions as they are primitive and universal reactions to outside stimuli and do not depend on cognitive processing or cultural environment. Even though there is some debate concerning the capacity of subjects with ASD to identify the basic facial emotions, there is more evidence to suggest that impairments of FER are constantly present in ASD (15–22). Recent literature reviews have shown important FER impairments in individuals with ASD (13, 23). Yet, several other studies did not replicate the same findings. This discrepancy was explained by different hypotheses. Indeed, some researchers suggested that FER in individuals with ASD is emotion-type-specific only manifesting for negative emotions such as fear (24, 25) and sadness (26). Other studies showed that these discrepant results may be explained by the use of tasks that lacked sensitivity to detect group differences. Lerner et al. (27) suggested that greater impairments in facial emotion recognition are present when adult facial expressions are presented to children with ASD, as opposed to child facial expressions. This hypothesis was supported by the study of Hauschild, indicating that adolescents, independently of ASD diagnostic status or severity of troubles had greater FER performance for child compared to adult faces. His findings suggested that “face processing abilities of adolescents with ASD may be influenced by experience with specific categories of stimuli, similar to their typically developing peers” (28). Therefore, we hypothesized that children with ASD would show greater performance in recognizing facial emotions of individuals having the same age range and that the assessment of facial emotion recognition could vary according to the age range of the model presented to the child.

The very mixed results on emotion recognition difficulties in ASD were also explained by “the use of very simplistic stimuli, i.e., “100% expression” or “full blown,” which may produce ceiling effects” (29–31). In fact, it was recently suggested that “individuals with ASD perceive exaggerated emotional facial expressions as being more representative of real-life emotions and more realistic” (32). Therefore, they may show more emotion recognition difficulties for low intensity facial expressions as subtle facial expressions afford less emotional cues to the observer (33). In the study of Wong et al. (34), “low intensity emotions” had also been associated with more important impairments in FER, particularly with negative emotions. Subtle or “low intensity” facial expressions are frequently observed in daily life. Even if children with ASD often show good performance in FER in laboratory setting, they are likely to have difficulties in the recognition of subtle facial expressions in real life. Indeed, it is possible that the lack of impairment of FER in individuals with ASD in some studies is explained by the abnormal processing of facial emotions, using high intensity of stimuli. In accordance with some authors, we suggested that difficult tasks with different intensity of facial emotions are needed to highlight emotion recognition difficulties in ASD (24, 35, 36).

We also suggested that the assessment of FER in individuals with ASD using videos or dynamic stimuli would show better performance. Our hypothesis was based on several research. In the study of Harwood et al. (37), authors have found that the use of “moving faces” to assess FER among individuals with intellectual disability may facilitate facial affect recognition, in comparison to static stimuli. Concerning typically developing individuals, “dynamic displays” were better recognized than “static displays” and judged as being more realistic and intense” (38). In the study of Ambadar et al. (39), dynamic stimuli had facilitated the recognition of subtle facial expressions. In the neuroimaging research, differential patterns of brain activation in response to dynamic in comparison with static facial emotions have been identified (40). As naturally facial emotions are intrinsically dynamic, authors suggested that dynamic stimuli may have a better ecological validity than static stimuli, as they may activate richer neuronal, automatic, and behavioral consequences than static stimuli (40, 41).

It is also important to note that, to date, only a few studies investigating emotion recognition in people with ASD have included an assessment of neutral facial expressions (25, 36, 42). Neutral facial expressions are frequent and important in everyday life and could represent a source of confusion for individuals with ASD.

Additionally, only a few studies on misinterpretation of emotions have used a combination of static and dynamic test, including images and videos, and, to the best of our knowledge, none has used tests including a combination of children, adolescent and adult facial expressions with different levels of intensity of facial emotions.

Therefore, and in order to better understand FER impairments in ASD that could conduct us to the creation of intervention tools, we chose to use an innovating task that takes into account all of the aforementioned limitations of previous studies. The current study is unique in that we used a recently developed and validated test, composed of a combination of static (pictures) and dynamic (videos) subtests, including children, adolescent, and adult faces, with high, medium, and low intensity of basic facial emotions, and neutral facial expression.

We hypothesized that:

(1) ASD group would show deficits in FER compared to control, expected mainly for negative emotions
(2) Performance in FER would vary according to the intensity of facial expression, with greatest deficits at lower intensities
(3) Deficits in the FER would be predominant in one type of support (video or pictures) with some suggestion that ASD group might show greatest performances with dynamic support, rather than with static images, with better performance in the identification of children's facial emotions.

## Materials and Methods

### Participants

Data in our study were collected from children with ASD and neurotypical children (NT) and all included children were attending ordinary schools. Participants in the clinic sample were recruited from the Department of Child and Adolescent Psychiatry in Razi Hospital, Tunis, Tunisia. The sample consisted of 45 Tunisian verbal children, aged 7–12 years (mean age = 9.26, sex ratio = 6.5), diagnosed with autism spectrum disorder, according to the Diagnostic and Statistical Manuel of mental disorders- fifth edition (DSM-5) criteria, after a full assessment by a child psychiatrist. This diagnosis was confirmed by a trained rater who administered the Autism Diagnostic Interview- Revised (ADI-R) (43) (Mean scores were: social interaction = 13,60 ± 1.64; verbal communication = 15,09 ± 3,44; restricted and stereotyped behavior = 5.7 ± 1.25).

The control group (neurotypical or NT children) was recruited from several primary schools and consisted of 117 Tunisian typically developing children, aged 7–12 years (mean age 9.22, sex ratio = 0.88). They had no history of psychiatric disease, neurodevelopmental disorder, and no family history of autism spectrum disorder. Children were assessed with the Mini International Neuropsychiatric Interview for Children and Adolescents (MINI-KID) (44) and those who had a present or a past psychiatric disorder were not included in the study.

All cases in both groups had a performance IQ >80. The fixed IQ cutoff was chosen so that to avoid the effects of deficits in receptive language and to select verbal children with ASD, who could be able to respond reliably to the test. Mental age was measured by The Tunisian version of the Differential scales of intellectual efficiency (EDEI)-A in its reduced form: the scale I “Vocabulary B” for verbal intelligence and the scale IV “categorical analysis” for non-verbal intelligence (45).

Exclusion criteria for both groups included: intellectual disability, a history of a traumatic brain injury, or seizure disorders, neurological or sensory deficits, substance use disorders that could affect children's cognitive functioning and children's performance during the test.

All the parents of selected children were invited to take part in the study, and provided written informed consent. The present study adhered to the tenets of the Declaration of Helsinki (2000).

### Measures

The REF task is a Tunisian validated and computerized test. It consists on a downloadable application on Android, developed by child psychiatrists and psychologists working in the Department of Child and Adolescent Psychiatry in Razi Hospital (Tunis, Tunisia). It is a part of a Tunisian battery for the assessment of social cognition (non-verbal and verbal theory of mind, empathy, and FER).

The REF task is an innovating test, having good psychometric properties (in review in the journal Frontiers in Psychology, 15 jan 2021, Taamallah, A., Halayem, S., Rajhi, O., Ghazzai, M., Moussa, A., Touati, M., et al. Validation of the Tunisian test for facial emotion recognition: study in children from 7 to 12 years).

The test consists of a combination of a static and a dynamic subtest. The static subtest includes 114 photographs of actors mimicking six basic emotions (happiness, disgust, fear, surprise, sadness, and anger) and neutral expression with three levels of intensity of facial emotions for the six basic emotions: low, medium, and high.

The dynamic subtest includes 36 videos with actors mimicking six basic emotions.

There were three male and three female actors having three age ranges: children, adolescents, and adults. Each actor displayed the six basic emotions, as well as a neutral face. The task includes photos and videos with an equal number of faces for each gender, age range, emotions type, as well as an equal number of intensities for each facial emotion in the static subtest. The duration of each video varied between 3 and 5 s with facial expression moving from neutral facial expression to the basic emotion proposed.

The test items were coded according to Ekman's “Facial Action Coding System” (FACS) method, comparing each photograph to that of neutrality. Depending on the FACS system, facial muscle contractions are coded in units of action (AUs). The nomenclature includes 46 AUs identified by a number. For example, AU1 corresponds to the “inner brow raiser.” For happiness, the units of action involved are AU6 (cheek raiser), AU12 (lip corner puller), and AU 25 (lips part). The intensity of the emotion depends on the number of AUs and the intensity of the contraction (46, 47). A professional photograph made the photos and the videos, and the recording sessions took place in a theater club.

**Figure 1:**
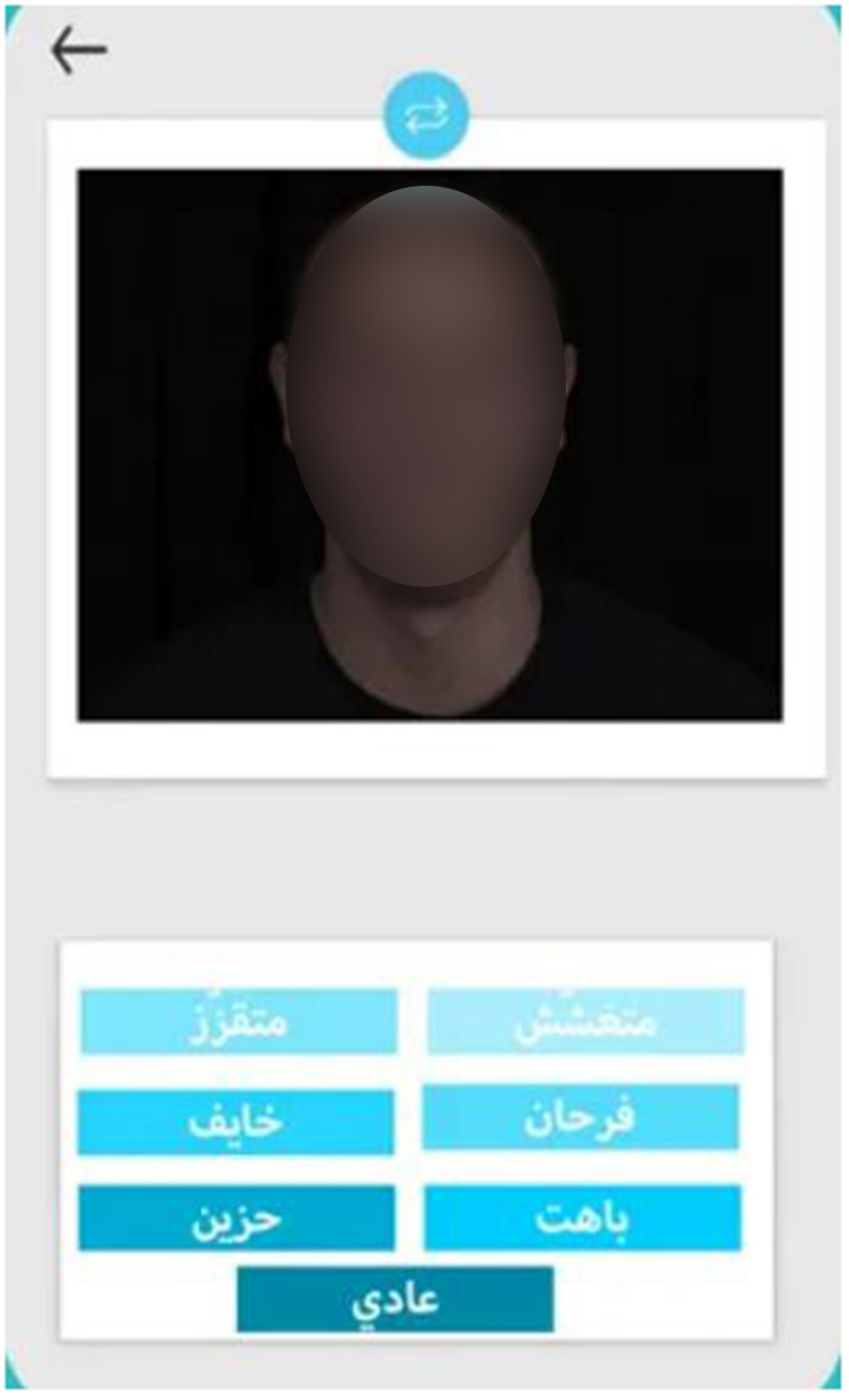
Interface of the REF task.

The interface was made up of two parts: the upper part displayed the photo or video, and the lower part displayed seven choices corresponding to the seven emotions (six basic emotions and neutrality) written in Tunisian Arabic dialect (Figure 1).

Stimuli are presented in a random and pre-established order and the whole face was displayed each time. For videos, and after the clip is played, the static image of the final intensity remains on the screen until the forced choice is made.

The distance between the child and the screen was set as 40 cm. Children were provided with standardized instructions and supervised by a trained- researcher. Before initiating the assessment, all participants (NT children and children with ASD) were invited to read the seven choices of facial expressions (happiness, disgust, fear, surprise, sadness, and anger) to confirm that they could correctly recognize them. Then, children were asked to view each face and to identify and select the facial emotion that corresponds to each photo or video.

For all participants, we started by presenting the static part of the test, then the dynamic one.

During the task, each proposed facial expression with the proposals remains on the screen 15 s. After this time and when the children were not able to select his choice, the next stimuli were presented. Once a response is chosen for a given face, participants are not able to revise it, and the next proposal of facial emotion is presented.

The duration of the test varied between 20 and 40 min depending on the child's performance and his response time.

### Data Analysis

Statistical analysis was performed using the Statistical Package for the Social Science (SPSS) version 26 for Windows. For descriptive statistics, we calculated the mean and the standard deviation for each of our continuous variables. For each of the continuous variables, normality was checked using Shapiro-Wilk's test. Indeed, according to the central limit theorem, a sampling distribution in samples larger than 30 tends to be normal, regardless of the shape of the data (48).

With large enough sample sizes (>30 or 40), the violation of the normality assumption should not cause major problems (4); this implies that we can use parametric procedures even when the data are not normally distributed.

To compare the sex distribution between groups, we used Pearson's Chi-square. To compare age, mental age, and school grade between the ASD group and the control group, we used Student's *t*-test for independent samples. To compare subscores within the same groups, we used the paired-samples *t*-test.

To examine the associations between the total scores for each of the static and dynamic subtests and age, mental age, and school grade, we used non-parametric Spearman's correlations.

To compare FER between ASD children and NT children, controlling for the effects of covariates (age, gender, mental age, and school grade) on emotion recognition scores, we chose to compare each of the subscale scores between groups using a separate univariate analysis of covariance (ANCOVA), and we adjusted the *p*-values to account for multiple comparisons. Since the number of subscores would make for too many dependent variables, it was more logical to run multiple separate univariate ANCOVA analyses than one single multivariate analysis of covariance (MANCOVA) analysis. Effect size was estimated using partial eta squares (η^2^). We checked for variance homogeneity using the Levene's test and for linearity and homogeneity of regression slopes using scatterplots (plotting each of the FER subscores as separate dependent variables against the each of covariates, for each subgroup).

Holm-Bonferroni's correction was applied in case of multiple comparisons.

A significance level of *p* = 0.05 was applied for all statistical tests.

## Results

Table 1 summarizes the general characteristics of the ASD group (*n* = 45) in comparison with the NT group (*n* = 117). While the mean age was comparable between groups, the ASD groups had a higher proportion of males, a lower mental age, and tended to have fewer years of study.

**Table 1:** General characteristics of the autism spectrum disorder group vs. the neurotypical group.

		**ASD group**	**NT Group**	***p***
		***n* = 45**	***n* = 117**	
Sex, *n* (%)	Male	39 (86.7%)	55 (47.0%)	<0.001
	Female	6 (13.3%)	62 (53.0%)	
Age, m ± SD		9.6 ± 1.5	9.2 ± 1.5	0.218
EDEI- Verbal mental age, m ± SD		8.7 ± 1.2	9.9 ± 0.9	<0.001
EDEI- Non verbal mental age, m ± SD		9.9 ± 2	10.9 ± 2.5	
EDEI- Mental age, m ± SD		9.3 ± 1.6	10.4 ± 1.7	
School grade, m ± SD		2.9 ± 1.4	3.5 ± 1.4	0.024

*ASD, Autism spectrum disorder; NT, Neurotypical*.

*m, mean; SD, standard deviation; EDEI, The Differential Scales of Intellectual Efficiency*.

In the ASD group, we found that the total score of the static subtest was positively correlated with school grade (rho = 0.404, *p* = 0.009), but not with age, or mental age. Similarly, the total score of the dynamic subtest was positively correlated with school grade (rho = 0.394, *p* = 0.012), but not with age or mental age. Both total scores for the static and dynamic subtests did not differ significantly between genders.

In the NT group, we found that the total score of the static subtest was positively correlated with age, mental age, and school grade (rho = 0.374, rho = 0.376, and rho = 0.394.; *p* < 0.001 for each correlation). Similarly, the total score of the dynamic subtest was positively correlated with age, mental age, and school grade (rho = 0.414, rho = 0.318, and rho = 0.434.; *p* < 0.001 for each correlation). Both total scores for the static and dynamic subtests did not differ significantly between genders.

After adjusting for sex, age, mental age, and years of study, the ASD group scored lower than controls on all tests except for the identification of fear and happiness in the static subtest, and the identification of happiness, fear, and sadness in the dynamic subtest (Table 2).

**Table 2:** One-way analysis of covariance comparing performances in facial emotion recognition in children with autism spectrum disorder vs. neurotypical children, controlling for sex, age, mental age, and years of study.

		**Static subtest**			**Dynamic subtest**		
		***F^a^***	***p^a^***	*****η^2^*****	***F^a^***	***p^a^***	*****η^2^*****
Total score		57.207	0.000	0.300	29.984	0.000	0.195
Score per emotion type	Anger	20.805	0.000	0.099	22.711	0.000	0.156
	Disgust	23.083	0.000	0.174	28.198	0.000	0.162
	Happiness	1.212	0.448	0.021	1.495	0.448	0.006
	Fear	3.525	0.189	0.018	4.140	0.176	0.033
	Surprise	47.614	0.000	0.286	10.659	0.009	0.053
	Sadness	17.108	0.000	0.125	5.666	0.095	0.050
	Neutral	10.341	0.012	0.086	51.204	0.000	0.271
Score per gender of the actors	Male	41.858	0.000	0.246	38.132	0.000	0.217
	Female	61.113	0.000	0.300	12.084	0.008	0.081
Pictures per age range of the actors	Children	34.978	0.000	0.307	11.109	0.000	0.132
	Adolescents	62.240	0.000	0.213	18.637	0.008	0.069
	Adults	40.810	0.000	0.239	38.959	0.000	0.207
Pictures per emotion's intensity	Low	53.118	0.000	0.280			
	Medium	46.313	0.000	0.257			
	High	45.153	0.000	0.255			

a *Adjusted for sex, age, mental age, and years of study*.

*p-values were adjusted for multiple comparison using Holm-Bonferroni's method*.

Out of the different six basic emotions, both groups (ASD and NT) scored best in identifying happiness on photos as well as in videos (*p* < 0.001 when compared with any of the other emotions).

In the ASD group, there was no significant differences in FER scores based on the actor's gender for photos, but scores were higher in the video tests when the actor was female (10.33 ± 3.89 vs. 8.65 ± 3.57, *p* < 0.001).

It was also easier for individuals with ASD to accurately identify facial emotions in children than in adolescents on photos and videos (17.17 ± 7.07 vs. 15.48 ± 6.79, *p* = 0.004; and 7.16 ± 2.69 vs. 5.70 ± 2.46, *p* < 0.001, respectively). However, identifying facial emotions were easier in children than in adults in videos (7.16 ± 2.69 vs. 6.12 ± 2.44, *p* < 0.001), but not on photos.

ASD children had more difficulties recognizing low-intensity than medium-intensity emotions (14.19 ± 6.80 vs. 15.60 ± 6.51, *p* < 0.001) and exhibited more problems identifying medium-intensity than high-intensity emotions (15.60 ± 6.51 vs. 17.50 ± 7.08, *p* = < 0.001).

## Discussion

Difficulty in FER is one of the most frequently identified social-cognitive impairments in individuals with autism spectrum disorder (23). However, it is still unclear if all facial emotions are equally impaired or whether recognition of some emotions could be impaired to a lesser extent or spared (13). In the present study, significant impairments in the FER performances were observed. After adjusting for sex, age, mental age, and years of study, children with ASD have shown impairment in the identification of anger, disgust, surprise, sadness, and neutral expressions. However, the recognition of fear and happiness didn't vary between the two groups. In addition, happiness was the easiest facial emotion to recognize for ASD and NT children.

In neurological terms, several studies have suggested that the processing of fear and negative emotions was linked to the amygdala functioning (49–54). Therefore, atypical function of the amygdala in autism spectrum conditions could lead to poor identification of negative emotions (55–57), which is partly compatible with our findings. Additionally, “dysfunction of the amygdala in autism could be the cause of a lack of orienting to the eyes in a face” (58, 59). This hypothesis has been mentioned in several studies that have found that individuals with ASD focus more on the lower part (i.e., the mouth), than the upper part (e.g., the eyes) of the face in identifying facial emotions (26, 60–64). In fact, individuals with ASD may find it less complicated to identify happiness, as focusing of the lower part of the face could be sufficient to recognize this emotion (65, 66).

Concerning the recognition of surprise, Baron-Cohen et al. (67) have suggested that specific impairment in recognition of surprise could be explained by impairment in the theory of mind in individuals with autism. In fact, the only basic emotion that needs judgment of another person's mental state is surprise. Consequently, if there is impairment in mental state judgments in autism, recognition of surprise would be altered (67). Thereby, further research studying the link between FER (specially surprise) and theory of mind is needed.

Recent studies have shown that other social and emotional dysfunctions in ASD, such as such as alexithymia (68), poorer social skills (69), living skills (70), social motivation for peer interaction (71–73), communication (74), and social functioning (75) may negatively impact FER. In our study, we didn't study these impairments due to the lack of contextualized and adapted tools.

From another point of view, impairment in FER for children with ASD could also be explained by the limited time for answer since they were not given ample time to respond. Several research reported that people with autism might be slower in recognizing facial emotion and can accurately recognize basic emotions, with no difference in performance between autistic and neurotypical people when they are given enough time to select their choice [e.g., (29, 76–78)].

Furthermore, these impairments may be due to the fact that individuals with ASD need more environmental or verbal contents, to identify accurately the facial emotion. Understanding emotions usually requires multi-sensory processing based on speech prosody and body and facial gestures (79–81).

Our study did not show a significant effect of age on FER performances in children with ASD and the accuracy of the FER was not positively associated with mental age. A recent meta-analysis, comparing FER in three age ranges (children, adolescents, and adults) has shown that “ASD was associated with deficits in FER across multiple expressions, and that these deficits were more pronounced in adults than in adolescents than in children and cannot be accounted for by intelligence” (12). The age range of our clinical sample (7–12 years) and the fact that we did not include adolescents and adults could explain our findings since younger subjects (children) with ASD seem to have better performances in FER than older subjects with ASD (adolescents and adults).

We also found that performances in FER (the total score of both static and dynamic subtest) were positively correlated with school grade in both groups. Our findings are in part consistent with those of Feinman and Entwisle. In their study, FER were assessed for children attending school and the study showed that FER increased significantly with each grade but leveled off between 8 and 11 years (82). These findings could be explained by the positive impact of the duration of social contact with peers that may improve emotion recognition and social skills for NT children and children with ASD.

In the present research, we used a combination of a static and a dynamic support. As the dynamic support could add ecological validity to the test compared with static support (40, 41), we hypothesized that children with ASD might show greater performance in dynamic subtest rather than static subtest. The ASD group performed worse than the NT sample in the static and dynamic subtest, with sadness being the only emotion that was better identified using the dynamic subtest, in comparison with the static subset. The dynamic subtest did not appear to improve FER considerably for children with ASD, in comparison with static stimuli. Thereby, our research did not support our hypothesis suggesting broad advantages associated with the use of dynamic stimuli. Our findings are consistent with those of Enticott et al. (83). Gepner et al. (84) suggested that children with ASD might show less impairments with slow dynamic displays of facial expressions. Studing FER in dynamic and realistic situations will be interesting for future research.

In both groups, we noticed a better FER of children's faces compared to those of adolescents and adults. Our findings are consistent of those of Lerner et al. (27), suggesting that more impairments in facial emotion recognition are found when adult facial expressions are presented to children with ASD, compared to child facial expressions. Thereby, the recognition of the same facial emotions, seen on age-peer faces could be better for both NT and ASD groups. Scherf et al. (85) suggested that “the own-age bias in face recognition, representing superior recognition abilities for faces of a similar age to the viewer, may emerge as a result of social reorientation toward peers during late-childhood and early-adolescence”. One recent study reported that healthy adolescents were more accurate in the recognition of the identity of individual faces having their age than the identity of adult faces. These findings suggested that FER performance could be moderated by the age range of facial stimuli (86). In contrast, Vetter et al. (87), suggested a lack of this moderating effect (for adolescents) in FER and reported similar accuracy in FER of adult and adolescent facial expressions in healthy adolescents. Thus, it seems possible that specifically children with ASD could exhibit greater difficulties identifying the facial emotions of people having different age ranges than their own, compared to adolescents and adults with ASD, and that the age of facial stimuli may moderate facial emotion perception for children with ASD. Thereby, further studies including and comparing facial stimuli of different age groups are needed in order to better specify the impact of the age range of facial stimuli on FER accuracy in individuals with ASD.

Performances on FER in the ASD group did not vary according to the gender of the actors in the static subtest, with a better performance in identifying facial emotions of female actors in the dynamic subtest. Our results may suggest that gender could affect the accuracy of FER in individuals with ASD with better performance in identifying facial emotions of female faces.

To date, none of all published studies has used pictures and videos of individuals of three age groups and different genders and none of them has compared performances on FER according to age and gender faces.

In our study, we assessed REF using three levels of facial emotion's intensity in the static subtest. For all intensities of emotions, the ASD group has shown worse performances compared to the NT sample. We also noticed that both groups were more accurate in identifying “high intensity” or “fully expressed” facial emotions with increasing difficulties at lower intensities. Our findings are in part consistent with previous recent findings: Wingenbach et al. (33), used a dynamic test with three levels of intensity of facial emotion and found that the ASD group had impairments in FER at low intensity, with greatest performance in identifying facial emotions at higher intensity.

Song and Hakoda (25) used morphing sequences of facial expressions with different intensities varying from 10 to 90% to study diminished sensitivity for FER recognition in children with autism spectrum disorder (14 ASD and 17 NT). They reported that the ASD group had higher thresholds than NT individuals for the recognition of facial emotions. In fact, at high intensities of facial emotions (e.g., 100%), all of the children with autism spectrum disorder were able to recognize facial expressions with the same accuracy of controls. However, as the facial emotions decreased in intensity, the FER accuracy in the ASD group was altered at a higher rate than that of the NT group. Authors suggested that people with autism spectrum disorder do not have a general, but a selective impairment in the recognition of basic emotions, limited to subtle and low intensity facial emotions and that they might use a compensatory mechanism that could help them to decode only high intensity facial emotion information (25). The differences between our results and those of the previously cited studies may be explained by the difference in power. Our study included a larger population, and thus had a better power to detect differences.

### Limitations

Despite the several strengths of the present study, several limitations need to be acknowledged. The limited number of female participants in the clinical sample did not allow us to perform significant statistical analyses of sex differences. In fact, a gender effect in the recognition of some facial emotion has been reported in neutrotypical individuals in a recent study (14). Furthermore, our ASD sample only consisted of children and our results might not be generalizable to adolescents and adults with ASD.

In addition, the large difference in sample size between the ASD group and the NT group is another limitation which may induce difficulties in constructing and interpreting the ANCOVA models. This difference in sample sizes can explained by the fact that the population of neurotypical children was used for the validation of the tool and that the recruitment of verbal children with ASD, verbal, attending school and having normal intelligence was more difficult than that of the control group. To ensure the assumptions of the ANCOVA tests are met despite the different sample sizes, we checked for variance homogeneity using the Levene's test and for linearity and homogeneity of regression slopes using scatterplots (plotting each of the FER subscores as separate dependent variables against the each of covariates, for each subgroup).

Finally, we did not match the two groups by gender or IQ. We included age, gender and mental age as covariates in the ANCOVA analyses to control for their potential effects on FER scores. The choice of not matching groups by IQ was also based on the results of two recent meta-analyses that have shown that IQ had no impact on FER performances in children with ASD (12, 13). We did not match the controls to the ASD cases by gender, since previous studies did not show that gender was independently associated with performance in FER tasks (83).

## Conclusion

Our study supports the findings that children with ASD have difficulties in FER. The ASD group has shown impairment in the recognition of all facial emotions except happiness and fear, with less pronounced difficulties for higher intensities of facial emotions and in the recognition of children facial emotions, compared to adolescents and adults' facial emotions. To the best of our knowledge, our study is the first one that used a test composed of a combination of static and dynamic subtests, including children, adolescent, and adult faces of males and females with high, low, and medium intensities of basic facial emotions, and neutral facial expression. Impairment in FER associated to ASD would have important negative impact in children's development, education, and social integration. Thus, it seems necessary to create new intervention instruments aiming to improve emotion recognition strategies in children with ASD at an early stage of development.

## Data Availability Statement

The original contributions presented in the study are included in the article/supplementary material, further inquiries can be directed to the corresponding authors.

## Ethics Statement

The studies involving human participants were reviewed and approved by Porfesseur Rym Ghachem, Razi Hospital, Manouba, Tunisia. Written informed consent to participate in this study was provided by the participants' legal guardian/next of kin.

## Author Contributions

SJ: elaboration of the research protocol, assessment of the ASD group, statistical analysis, and redaction of the article. SH: elaboration of the research protocol and correction of the article. AT: elaboration of the facial emotion recognition test and its administration for the control group. SE: assessment of the ASD group. OR, MG, and MM: administration of the test for the control group. AN, ZA, and MH: elaboration of the test. SO: statistical analysis and correction of the article. RF: statistical analysis. AB: elaboration of the test and of the research protocol and correction of the article. All authors contributed to the article and approved the submitted version.

## Conflict of Interest

The authors declare that the research was conducted in the absence of any commercial or financial relationships that could be construed as a potential conflict of interest.

## Publisher's Note

All claims expressed in this article are solely those of the authors and do not necessarily represent those of their affiliated organizations, or those of the publisher, the editors and the reviewers. Any product that may be evaluated in this article, or claim that may be made by its manufacturer, is not guaranteed or endorsed by the publisher.
